# Cheonggukjang-Specific Component 1,3-Diphenyl-2-Propanone as a Novel PPARα/γ Dual Agonist: An In Vitro and In Silico Study

**DOI:** 10.3390/ijms221910884

**Published:** 2021-10-08

**Authors:** Radha Arulkumar, Hee-Jin Jung, Sang-Gyun Noh, Daeui Park, Hae-Young Chung

**Affiliations:** 1 Interdisciplinary Research Program of Bioinformatics and Longevity Science, Pusan National University, Busan 46241, Korea; radhuspn@gmail.com (R.A.); rskrsk92@naver.com (S.-G.N.); 2 Department of Pharmacy, College of Pharmacy, Pusan National University, Busan 46241, Korea; 3 Department of Predictive Toxicology, Korea Institute of Toxicology, Daejeon 34114, Korea; daeui.park@kitox.re.kr

**Keywords:** Cheonggukjang volatile compounds, fermented soybean, molecular docking, PPARα/γ dual agonist, 1,3-diphenyl-2-propanone

## Abstract

Background: Cheonggukjang is a traditional fermented soybean paste that is mostly consumed in Korea. However, the biological activities of Cheonggukjang specific compounds have not been studied. Thus, we aimed to discover a novel dual agonist for PPARα/γ from dietary sources such as Cheonggukjang specific volatile compounds and explore the potential role of PPARα/γ dual agonists using in vitro and in silico tools. Methods: A total of 35 compounds were selected from non-fermented and fermented soybean products cultured with *Bacillus subtilis*, namely Cheonggukjang, for analysis by in vitro and in silico studies. Results: Molecular docking results showed that 1,3-diphenyl-2-propanone (DPP) had the lowest docking score for activating PPARα (1K7L) and PPARγ (3DZY) with non-toxic effects. Moreover, DPP significantly increased the transcriptional activities of both PPARα and PPARγ and highly activated its expression in Ac2F liver cells, in vitro. Here, we demonstrated for the first time that DPP can act as a dual agonist of PPARα/γ using in vitro and in silico tools. Conclusions: The Cheonggukjang-specific compound DPP could be a novel PPARα/γ dual agonist and it is warranted to determine the therapeutic potential of PPARα/γ activation by dietary intervention and/or supplementation in the treatment of metabolic disorders without causing any adverse effects.

## 1. Introduction

Soybean is a functional dietary dish in Asian countries such as Japan and Korea because of its rich protein and oil contents [1]. Fermented soybeans have higher nutritional components than non-fermented soybeans and are easily digestible. Cheonggukjang (CGJ) is a commonly consumed fermented soybean paste in South Korea [2,3], which may enhance immune activity, inhibit murine allergic asthma, regulate lipid metabolism, and fight against neurodegenerative diseases [4,5,6,7]. CGJ is a steamed fermented soybean manufactured using *Bacillus subtilis* culture that can produce various bioactive constituents, including organic acids, amino acids, fatty acids, and volatile compounds [3]. Recently, volatile compound and fatty acid profiles during CGJ fermentation have been reported [2]. However, the biological activities of CGJ-specific volatile compounds in age-related metabolic disorders and their underlying mechanisms have not been studied.

Peroxisome proliferator-activated receptors (PPARs) are ligand-dependent intracellular proteins that act as transcription factors by binding to specific DNA sequences of appropriate genes and stimulating transcription activity upon ligand activation. The activated transcription factors are mainly involved in cellular differentiation, development, metabolism, inflammation, and tumorigenesis [8,9,10]. There are three PPAR subtypes: PPARα, PPARγ, and PPARβ/δ. Generally, PPARs heterodimerize with another nuclear receptor, the retinoid X receptor (RXR), and the PPAR-RXR complex was translocated into the nucleus, where it can bind to peroxisome proliferator hormone response elements (PPREs) with the promotor of the target DNA [10]. A heterodimer complex recruits a transcription coactivator when activated by an agonist and controls the transcription of genes, which regulate the lipid and carbohydrate digestion systems. PPARα is highly expressed in many tissues with a higher capacity for fatty acid oxidation, such as the liver, kidney, and heart muscle; it regulates the genes involved in lipid catabolism [8,9,10]. PPARα activation can increase the high-density lipoprotein (HDL) cholesterol synthesis and cholesterol transport and reduce the triglyceride levels [11,12,13]. Similarly, PPARγ plays a pivotal role in cellular proliferation and differentiation, stimulating lipid storage, and subsequently improving insulin sensitivity indirectly, and augmenting glucose disposal in adipose tissues and skeletal muscles [13]. Moreover, improving insulin sensitivity and increasing HDL levels through ligand activation of PPARβ/δ has been reported to be a potential target in the treatment of obesity and dyslipidemias [14]. Although PPARγ agonists from thiazolidinedione have been used clinically, they have serious side effects [15,16,17,18]. Elafibranor and GFT505, which act as dual PPARα and PPARδ agonists, have shown a good response in the treatment of non-alcoholic steatohepatitis/non-alcoholic fatty liver disease (NASH/NAFLD) and its associated metabolic syndrome (MetS) [19,20]. Elafibranor has been reported to exert favorable effects on glucose levels, lipid profiles, liver enzymes, and the inflammatory response in patients with NASH; however, it failed to alleviate hepatic fibrosis in phase 3 clinical trials [21]. Thus, the discovery of agonists from natural herbs and dietary sources for the activation of PPARs could be useful for improving lipid metabolism and insulin sensitivity and tackling aging and cancer, without causing any adverse effects.

In this study, we aimed to explore the biological activities of CGJ-specific volatile compounds using in vitro and in silico tools. Thus far, 35 compounds have been screened and docked with various molecular targets. Among them, six compounds showed lower docking scores for PPARα and PPARγ. More specifically, 1,3-diphenyl-2-propanone (DPP) had the lowest docking score for activating PPARα (1K7L) and PPARγ (3DZY); the in silico approach was used to analyse the physicochemical and pharmacokinetic properties of the chosen compounds using the ProTox-II and PreADMET servers. Furthermore, the effect of DPP on PPARα/γ activation in Ac2F liver cells was examined. These results suggest that the CGJ-specific compound DPP is a novel PPARα/γ dual agonist.

## 2. Results

### 2.1. In Silico Screening of Volatile Compounds from Cheonggukjang by Culturing with B. subtilis

**Figure 1:**
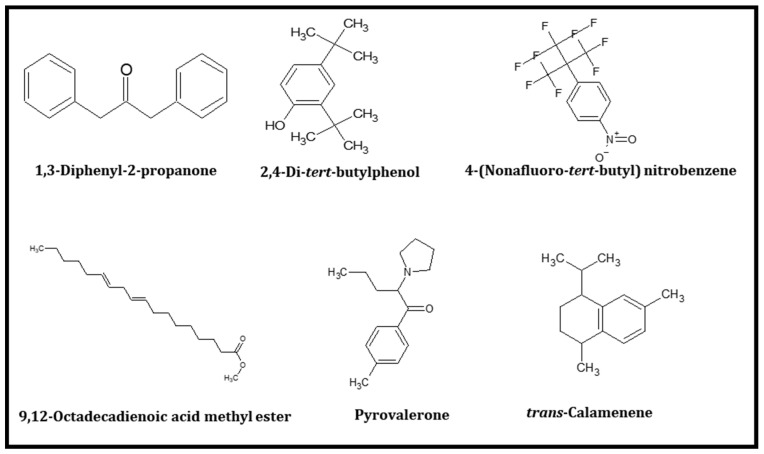
Structures of six best score volatile compounds from fermented soybean Cheonggukjang.

Chukeatirote et al. (2017) [22] identified 67 volatile compounds from non-fermented and fermented soybean products cultured with *B. subtilis*. Among them, 35 volatiles were identified only in fermented soybean products, including seven alcohols, five aldehydes, one aromatic, five ketones, nine acids and esters, four pyrazines, and four miscellaneous compounds (Table 1). Thirty-five compounds were selected for further analysis and screening by conducting in vitro and in silico studies. Molecular docking studies of 35 volatiles were performed to investigate their binding status to the active site of 10 distinctive proteins, namely PPARα (1K7L), PPARβ (1GWX), PPARγ (3DZY), AMPK(2Y94), LKB1(2WTK), PAR2 (modelled), SIRT1(4I5I,) SIRT2(5YQL), SIRT3 (4BN5), and SIRT6 (3K35). A total of 35 compounds were tested for distinctive proteins, while six compounds with a lower docking score (Table 2, Table 3 and Table 4), and high-affinity binding were found. The six compounds were 1,3-diphenyl-2-propanone, 2.4-di-*tert*-butylphenol, 4-(nonafluoro-*tert*-butyl)-nitrobenzene, 9,12-octadecadienoic acid methyl ester, pyrovalerone, and *trans*-calamenene. The chemical structures of these six volatiles are shown in Figure 1.

### 2.2. Molecular Docking Study of DPP as a PPARs Agonist 

**Figure 2:**
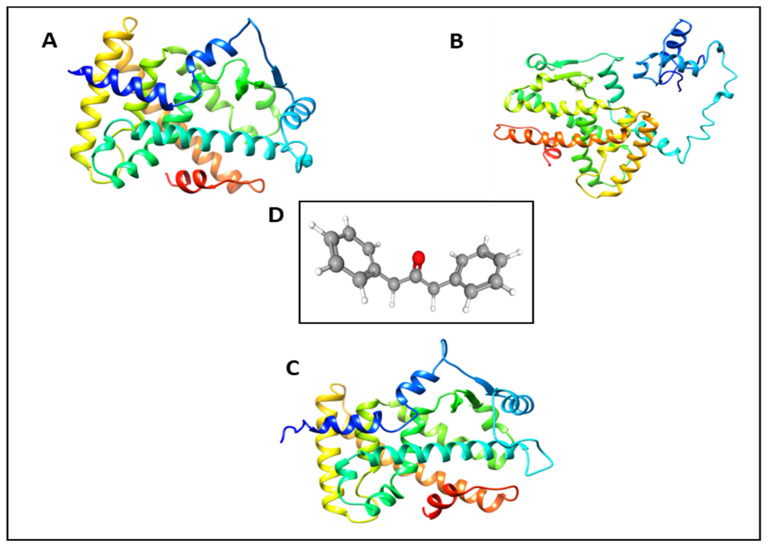
Crystalline structure of receptor (**A**) PPARγ-3DZY (**B**) PPARα-1k7l A (**C**) PPARβ-1GWX A http://rcsb.org (accessed on 27 September 2019) (**D**) Optimized structure of small compound 1,3-Diphenyl-2-propanone.

As previously mentioned, PPARs are associated with cellular mechanisms, such as differentiation, development, metabolism, and tumorigenesis [8,9,10]. Molecular docking tools have been reported to be useful for detecting potential drug candidates based on their affinity for binding to target proteins [23]. Moreover, the lower affinity value of binding indicates a higher possibility of binding to the target protein [24,25]. The binding energy was negative. Thus, the change (ΔG) indicates that the binding process is spontaneous and the drug fits well in the binding pocket receptor, forming the most stable drug receptor [26]. If the binding energy value of the chemical compound is negative and larger, accepted as a drug [27]. In this study, an in-silico docking simulation was carried out using AutoDock Vina, AutoDock 4.2.1, and Dock6. The results of molecular docking showed that 1,3-diphenyl-2-propanone (DPP) had lower docking PPARα (1K7L), PPARꞵ (1GWX) and PPARγ (3DZY) scores than the positive controls (Figure 2) than that of AMPK (2Y94), LKB1 (2WTK), PAR2 (modelled), SIRT1(4I5I,) SIRT2 (5YQL), SIRT3 (4BN5), and SIRT6 (3K35). Hence, we have selected PPARs for further analysis and, hypothesized that DPP can be a PPARs agonist.

The docking scores of 1,3-diphenyl-2-propanone were −8.8, −8.14, and −30.4204 (AutoDock Vina, AutoDock 4.2.1, and Dock6) for PPARα; −7.5, −7.36, and −34.8391 (AutoDock Vina, AutoDock 4.2.1, and Dock6) for PPARβ; and −7.0, −7.53, and −36.0086 (AutoDock Vina, AutoDock 4.2.1, and Dock6) for PPARγ. The binding energy and interacting residues obtained from PPARα, PPARβ, and PPARγ with DPP molecular docking calculations are listed in Table 5, Table 6 and Table 7. DPP appeared to have lower scores than the other compounds of CGJ volatiles. This finding indicates that DPP has the best affinity at the binding site of the anti-aging receptors 1K7L, 1GWX, and 3DZY of PPARα, PPARβ, and PPARγ, respectively.

### 2.3. Pharmacophore Validation of DPP

**Figure 3:**
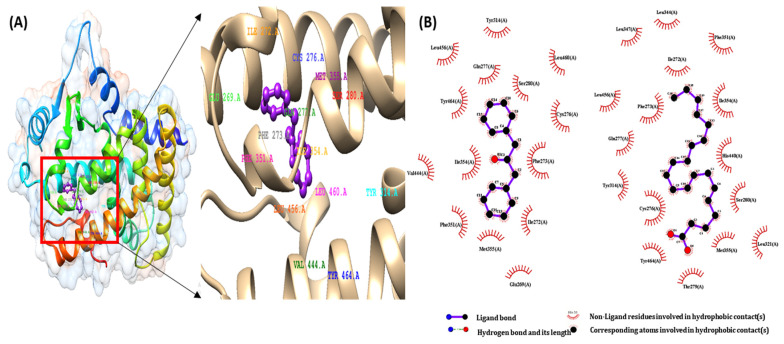
(**A**)The binding interactions of 1,3-Diphenyl-2-propanone ligand with PPARα protein (**B**) pharmacophore analysis between PPAR α and active components of 1,3-Diphenyl-2-propanone and PPARα- Eicosapentaenoic acid (control).

**Figure 4:**
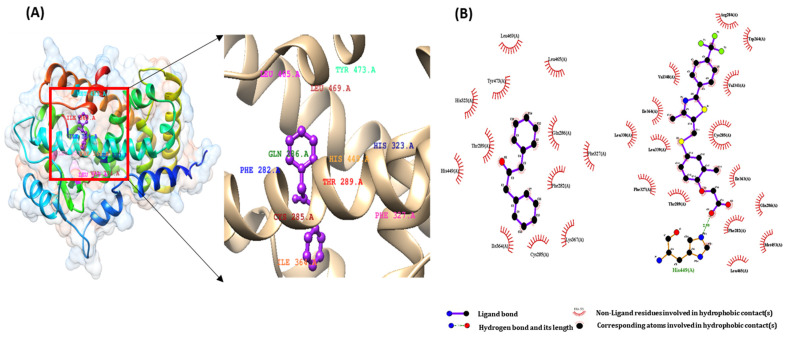
(**A**)The binding interactions of 1,3-Diphenyl-2-propanone ligand with PPARβ protein (**B**) pharmacophore analysis between PPAR β and active components of 1,3-Diphenyl-2-propanone and PPAR β-GW501516 (control).

**Figure 5:**
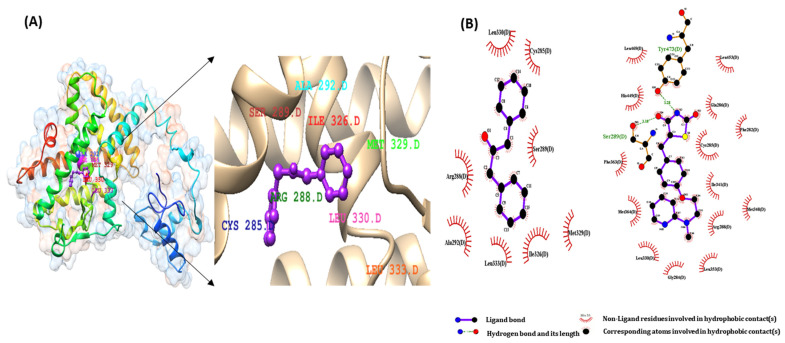
(**A**)The binding interactions of 1,3–Diphenyl-2-propanone ligand with PPARγ protein (**B**) pharmacophore analysis between PPARγ and active components of 1,3-Diphenyl-2-propanone and PPARγ-rosiglitazone (control).

The binding interactions of the first active docked compliance of the ligands of the CGJ volatile compound and the target proteins were recognized using the Ligplot+ tool. We checked all amino acids inside the active site of the target protein, and important binding interactions were recognized. With regard to hydrogen bonding, electronic bonding, hydrophobic interactions, and van der Waals interactions also influenced the activity of ligands inhibiting the receptor [28]. The interaction of ligands with PPARα-eicosapentaenoic acid (control), PPARβ-GW501516 (control), and PPARγ-rosiglitazone (control) and that of DPP with the binding pocket receptor are shown in Figure 3A, Figure 4A and Figure 5A.

As shown in Figure 3B, eicosapentaenoic acid and DPP occupied the same binding pocket of the 3DZY receptor with some active amino acid residues, that is, CYS276, GLN 277, SER 280, TYR 314, MET 355, LEU 456, and TYR 464. In Figure 4B, GW501516 and DPP occupied the same binding pocket of 1GWX receptor with some active amino acid residues, i.e., PHE 282, CYS 285, THR 289, ILE 364, and HIS 449; in the same way, rosiglitazone and DPP occupied the same binding pocket of 1K7L receptor with some active amino acid residues, i.e., CYS 285, ARG 288, SER 289, and LEU 330. The results of these interactions are shown in Figure 5B.

### 2.4. In Silico Toxicity Study of DPP

Determining the toxic effects of these compounds is important for selecting a candidate drug. Therefore, the toxic effects of the volatile compounds in this research were tested. During the test, the disposition of a pharmaceutical compound’s toxicity properties within the human body was determined. Because of poor toxic properties, most of the previously reported inhibitors are considered to be ineffective; hence, in the early stages of drug development, filtering and optimization of the toxicity properties are necessary to avoid treatment failure. An important criterion for choosing a therapeutic candidate is the toxicity of the compounds. Toxicity classes were defined based on the Globally Harmonized System of Classification and Labelling of Chemicals. LD_50_ values under Class VI were considered non-toxic (LD_50_ > 5000). Hence, we tested the toxicity of the six volatile compounds using the ProTox-II webserver (Table 8). Most of our volatile compounds, such as DPP, 2.4-di-*tert*-butylphenol, 4-(nonafluoro-*tert*-butyl) nitrobenzene, and pyrovalerone, have shown very low toxicity. More importantly, the active compound DPP was non-toxic.

### 2.5. ADMET Prediction of DPP

The most important method to develop a new drug molecule is to determine how an organism affects a drug. The SwissADME tool was used to calculate its drug-likeness properties based on the Lipinski’s and Veber’s rules (Table 9), while the PreADMET online software was used to estimate the pharmacokinetic properties (absorption, distribution, CYP inhibition, and substrate) (Table 10 and Table 11) of the volatile compound CGJ. The abovementioned program determines the pharmacokinetic properties such as BBB penetration human intestinal absorption (HIA%), CaCo-2 permeability, MDCK cell permeability, plasma protein binding (%), and skin permeability (log kp). The capacity to enter the BBB is a prerequisite for neurotherapeutic drugs. The online BBB permeability prediction of DPP yielded a score of 1.75109, which is significantly higher than the minimum required for BBB permeation (0.02) [29]. The compound complies with the Lipinski’s rule of five or Pfizer’s rule of five (Table 9) [30] and Veber’s rule of three [31]. These results suggest that the desired compound is an orally active drug candidate. As shown in Table 10, the respective ADME profiles of the selected candidate molecules were obtained from the PreADMET server.

The computational BBB permeability value was highest in the compound DPP, while the control molecules (PPAR α/γ) had a lower computational BBB permeability value. As explained above, the Caco-2 and HIA values indicate the intestinal absorption status. The absorption values predicted for Caco-2 cells (PCaco-2) were found to be between 4 and 70. Subsequently, the values of the compounds used in the investigation indicated moderate BBB permeability. The Caco-2 value was again highest for the DPP, among other test candidates, which was comparable to that of the control molecules eicosapentaenoic acid (PPARα) and rosiglitazone (PPARγ). Its HIA value was also the highest among the drug candidates and the control group. The compounds included in the study can be well absorbed through the intestinal cells, as the predicted HIA values for most of the compounds were found to be 100%. As described above, the MDCK computational component predicts the renal clearance of the molecule. As per the derived values, the MDCK value of the DPP was the best among the candidates. However, the MDCK value of DPP was higher than that of the control molecules eicosapentaenoic acid (PPARα) and rosiglitazone (PPARγ). The PPB indicates the plasma protein binding of the drug and predicts its retention in the system, as well as the resultant clearance. The Plasma protein binding (PPB) value of DPP was close to that of the control drugs, indicating that the compounds were strongly bound chemicals. Thus, from the different values derived, we can predict the best candidate among the drug compounds, which is one of the analogs of the candidate compound DPP. The results were compared with those of the control molecules eicosapentaenoic acid (PPARα) and rosiglitazone (PPARγ). We conclude that although DPP had a lower BBB permeability than a few of the test ligands and the control (PPARα), it had an HIA probability almost close to that of the control ligands. Apart from these, the LD_50_ value was in the non-toxic range for DPP; in terms of metabolism, DPP, which remains the best candidate compound to date, demonstrated an inhibition scene for CYP3A4, CYP2C9, and CYP2C19. This finding shows that DPP may inhibit these CYP450 isoforms, which may lead to an increase in those drugs that are the substrates of the three isoforms. However, since these are computational subtle advances in vivo, comparative investigations should be performed to determine all the in silico conceivable outcomes for metabolism.

### 2.6. Molecular Dynamics Simulation Analyses of DPP with PPARα/γ 

To analyse the trajectory of the protein-ligand complex during the dynamic process, we must validate the stability of docking results and calculate the binding free energies through the MD simulation process [32]. The flexibility of the DPP compound and the overall stability of the docking complexes we evaluated using the Gromacs 5.1.2 software package. The root mean square fluctuation (RMSF) and root-mean-square deviation (RMSD) graphs were generated using the Gnuplot software to determine the residual fluctuations and deviations of the active compound DPP and the control compound eicosapentaenoic acid. Based on the results of the MD simulations, possible hydrogen bond residues of DPP interactions with PPARα were selected. As mentioned in Table 12, GLN277 and HIS440 were identified as hydrogen-bond donor residues.

**Figure 6:**
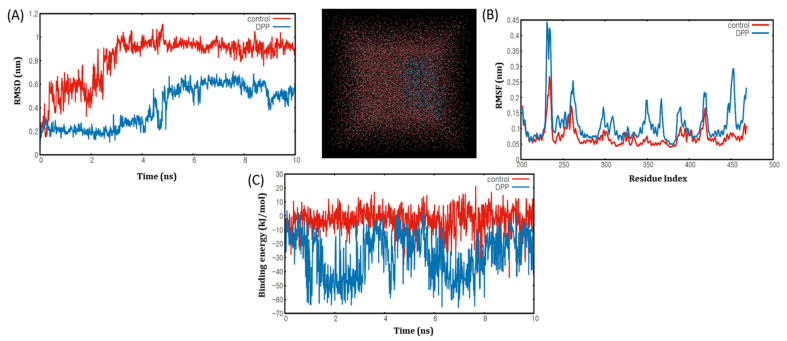
MD simulation analyses (**A**) root mean square deviation (RMSD) plot of PPARα-Eicosapentaenoic acid (control) and 1,3-Diphenyl-2-propanone (dpp) complex at10 ns of molecular dynamic simulation. (**B**) the RMSF plot of the back-bone heavy atoms of the Eicosapentaenoic acid (Red) and 1,3-Diphenyl-2-propanone (Blue) complex. (**C**) binding energy plot of Eicosapentaenoic acid (Red) and 1,3-Diphenyl-2-propanone (Blue) complex at10 ns.

As shown in (Figure 6A), the RMSD for the PPARα-DPP complex (blue) was eventually stable at around 0.2 nm and 0.6 nm during simulations. The backbone RMSD of DPP and eicosapentaenoic acid revealed that an equilibrated and converged state was achieved after 5 ns of simulation compared with the control. Therefore, MD simulations were extended to 10 ns to gauge the stability of these active compound binding systems. Furthermore, to estimate the flexibility of protein residues and verify the movement of amino acid residue binding to PPARα of DPP throughout the MD simulation, we plotted the RMSFs for carbon α-atoms of all residues. The RMSF plots of the PPARα-DPP complex (blue) and PPARα-eicosapentaenoic acid complex (red) were generated (Figure 6B).

We found that the RMSF curve was similar to that of the PPARα-DPP complex or PPARα-eicosapentaenoic acid complex, and no major fluctuations were observed. Therefore, the difference in RMSF values for some residues indicated that DPP binds tightly to the active site of PPARα. In this study, the binding free energy was calculated using the MM/PBSA method implemented in GROMACS version 5.1.2. A total of 2000 snapshots from the last 20 ns of the MD simulations of the complexes of DPP with PPARα were used to perform binding free energy (ΔGbinding) calculations (Figure 6C), and the results were as follows: Coiul-SR = −28.5083 kcal/mol and Lj-SR = −116.45 kcal/mol. Similarly, the best binding score for PPARγ-DPP (Figure S1 and Table S1) complex was further investigated through MD simulation, but the RMSD and RMSF values were not satisfactory. To the best of our knowledge, this is the only study that showed the RMSF distribution of PPARα-DPP, indicating that they can sufficiently fix most of the residues with control residues; the RMSD was stable, and the binding energy was also within the required limit. Therefore, these computational analysis results are in agreement with the inhibition of metabolic disorders associated genes presented in this study, demonstrating that DPP is a strong PPARα agonist.

### 2.7. Evaluation of Toxicity of Volatiles by an In Vitro Analysis

**Figure 7:**
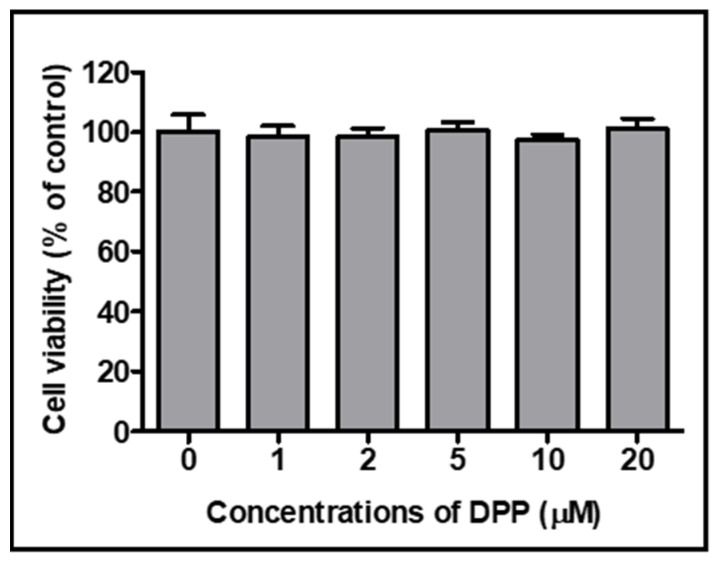
Cytotoxicity effect of DPP in Ac2F liver cells. Cells were cultured with the different concentrations of DPP (0–20 µM) for 24 h. Cell viability was assessed using the Ez-Cytox cell viability assay kit. Values are expressed as the mean ± SEM of three independent replications.

The MTT assay was performed to evaluate the cytotoxicity of DPP on rat Ac2F endothelial cells. The cells were treated with different concentrations of DPP (0–20 µM) and incubated for 24 h (Figure 7). Results demonstrated that an DPP level of 20 µM did not cause cytotoxicity in Ac2F cells. Hence, we used up to 10 µM of DPP in additional experiments, such as western blotting.

### 2.8. In Vitro Study of DPP as a PPARα/γ Dual Agonist 

**Figure 8:**
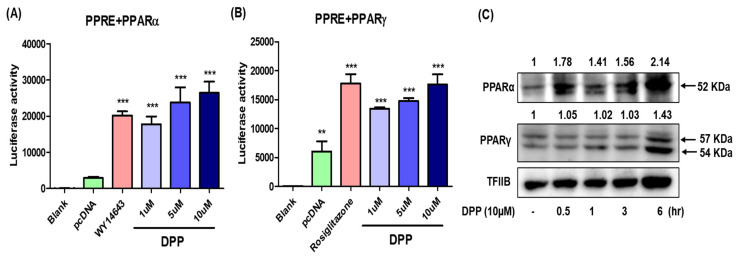
DPP increased transcriptional activity of PPARα and PPARγ. For luciferase, the 3× PPRE-TK-LUC plasmid and PPARα (**A**) or PPARγ (**B**) expression vector were transfected into Ac2F cells. Twenty-four hours after the transfection, the cells were treated with DPP or agonists (WY14643 10 µM and Rosiglitazone 10 µM, respectively) for 5 h. Values are expressed as the mean ± SEM of two independent replications. (**C**) The nuclear levels of PPARα and PPARγ in Ac2F cells were analysed by western blot and band intensity were quantified by ImageJ program. Transcription factor II B (TFIIB) was used as loading control. Statistical results of one-factor ANOVA followed by the Bonferroni test: ** *p* <0.05 and *** *p* < 0.001 vs. blank.

To confirm whether DPP activates PPARα and PPARγ activity, Ac2F cells were pre-treated with the PPARα and PPARγ agonists WY14643 and rosiglitazone, respectively, and a cell-based reporter gene assay was performed. We observed that DPP increased the transcriptional activities of PPARα and PPARγ in Ac2F cells compared to control (Figure 8A,B). Next, we examined whether DPP could activate PPARα and PPARγ in vitro using Ac2F cells. Western blotting was performed to measure the protein expression using DPP (10 µM)-treated Ac2F cells at different time intervals. The results showed that the nuclear level of PPARα in a time-dependent manner and PPARγ 6h after treatment with 10 µM of DPP (Figure 8C). These data suggest that DPP could be a more promising agonist of PPARα than PPARγ, which is consistent with the results of MD simulation analyses.

## 3. Discussion

In this study, we screened CGJ-specific volatile compounds and investigated their biological roles using in vitro and in silico tools. Among the screened volatile compounds, DPP had the lowest docking score for activating PPARα (1K7L) and PPARγ (3DZY), suggesting that DPP has the best affinity to the binding site of PPARα and PPARγ. Furthermore, DPP significantly increased the PPARα and PPARγ activities and activated the expression of PPARα and PPARγ in Ac2F cells. We also found that DPP binds tightly to the active sites of PPARα and eicosapentaenoic acid, and their RMSF curves were similar. In addition, the active compound DPP exhibited a lower toxicity. Our results demonstrate that the CGJ-specific compound DPP could be a novel PPARα/γ dual agonist and does not cause adverse effects.

Molecular docking tools have been widely used for the detection of potential drug candidates based on their affinity for binding to target proteins [24]. In addition, the lower affinity value of binding indicates a higher possibility of binding to the target protein [25,26]. In this study, we performed an in silico docking simulation using AutoDock Vina, Auto Dock4.2.1, and Dock6 with PPARs, such as PPARα, PPARγ, and PPARβ/δ, associated with cellular differentiation, development, metabolism, and tumorigenesis [8,9,10]. Among PPARs, PPARα specifically controls the expression of genes implicated in fatty acid oxidation and energy homeostasis [33]. The results showed that DPP had a lower docking score for PPARα (1K7L) and PPARγ (3DZY) than the positive controls (Figure 2). Interestingly, DPP showed the lowest score among the compounds derived from the CGJ volatiles. Previous studies have demonstrated a relationship between PPARα dysfunction and the aging process [34,35]. The expression or activity of PPARα was downregulated during the aging process in various tissues, such as the heart, kidney, and spleen [36,37,38]. PPARα−/− mice and hepatocyte-specific PPARα−/− mice exhibited malfunction in lipid metabolism during aging, which subsequently led to early hepatic steatosis [39]. Similarly, our previous study also demonstrated an impairment of PPARα in the regulation of age-associated renal fibrosis in aged PPARα−/− mouse models [40]. The results of the present study suggest that DPP had the lowest docking score for activating PPARα (1K7L), increased the PPARα activity, and activated the Ac2F cells in vitro (Figure 7A,C). In addition, MD stimulation results showing the high affinity of ligand compared control and the residues of the protein forming a stable complex with low flexibility (Figure 8). These findings indicate that DPP can activate PPARα, which is associated with alterations in lipid metabolism in MetS and aging.

Another PPAR subtype, PPARγ, plays an important role in lipid metabolism, improving insulin sensitivity, and augmenting glucose disposal in adipose tissues and skeletal muscles [13]. In addition, PPARγ agonists, namely, pioglitazone and rosiglitazone, are the currently approved drugs for the management of hyperglycaemia in patients with type 2 diabetes mellitus [41]. In addition, many PPARγ agonists are effective in lowering triglyceride levels in the plasma; regulating lipid accumulation in various tissues such as the liver, heart, and skeletal muscle; and controlling insulin sensitivity [42,43]. Hence, we performed an in-silico docking simulation with PPARγ and examined whether DPP can activate PPARγ in vitro using Ac2F cells. The docking results revealed that DPP has the best binding affinity for the anti-aging receptor 3DZY of PPARγ. Furthermore, the in silico physicochemical and pharmacokinetic properties indicate that lead compounds can be good predictors of nontoxicity and have good absorption, penetration, and permeability abilities in the human body, thus indicating high potential for BBB permeability, HIA, PPB, and Caco-2 permeability. Moreover, in vitro results showed that DPP increased PPARγ activity and triggered its expression in Ac2F cells compared to that in control cells (Figure 8B,C). These data suggest that DPP could be a PPARγ agonist; however, results of the MD simulation analyses showed the lower affinity of this ligand when compared with that of the control. In addition, elafibranor acts as a dual PPARα/δ agonist and improves the cardio metabolic risk profiles of patients with NASH [19]. Furthermore, elafibranor is still under phase 3 trials and is being tested in patients with moderate to severe symptoms. Our findings suggest that DPP could be a stronger agonist of PPARα than PPARγ, whose dysregulation is associated with MetS.

## 4. Materials and Methods

### 4.1. Tools

This study utilized a personalized computer with an Intel(R) Core (TM) i7 CPU, X86-64-bit, 6 GB RAM to illustrate the experiment. The software used were as follows: RCSB PDB (RRID: SCR_012820), NCBI PubChem (RRID:SCR_004284), AutoDock Vina v.1.1.2, (RRID:SCR_011958), AutoDock v.4.2.6, (RRID:SCR_012746), UCSF Dock v.6.7, UCSF Chimera v.1.14 (RRID: SCR_004097), Ligplot+ v.2.2. (RRID: SCR_018249), PreADMET, ProTox-II, GROMACS v.5.1.2, (RRID:SCR_014565), and Gnuplot v.5.4.2 (RRID:SCR_008619).

### 4.2. Materials

Thirty-five three-dimensional (3D) structures of CGJ compounds were discovered in our investigation [22], and a crystalline form of receptor with the following PDB Ids: 1K7L (PPARα), 1GWX (PPARβ), 3DZY (PPARγ) MPK (2Y94), LKB1 (2WTK), PAR2 (modelled), SIRT1 (4I5I,) SIRT2 (5YQL), SIRT3 (4BN5), and SIRT6 (3K35) were taken for the experiment.

### 4.3. Protein and Ligand Preparation

Protein and ligand arrangements were performed utilizing the UCSF Chimera software version 1.13.1 (construct 41965). Protein targets with PDB IDs 1K7L.pdb (PPARα), 1GWX.pdb (PPARβ), and 3DZY.pdb (PPARγ), 2Y94.pdb (AMPK), 2WTK.pdb (LKB1), PAR2.pdb (PAR2 modelled), 4I5I.pdb (SIRT1), 5YQL.pdb (SIRT2), 4BN5.pdb (SIRT3), and 3K35.pdb (SIRT6) were downloaded from the RCSB database, a Protein Data Bank powered by UCSF Chimera. Proteins and local ligands were separated and saved with the following filenames: protein.pdb. mol2, ligand.pdb, and ligand. mol2. Local ligands were diligently arranged, while new ligands (volatile compounds) were downloaded from the PubChem database https://pubchem.ncbi.nlm.nih.gov/ (accessed on 27 September 2019) to prepare conformations utilizing the Marvin Sketch software version 17.1.30 and saved using the filename ligand.pdb and ligand. mol2.

### 4.4. Docking Protocol Validation

The root mean square deviation (RMSD) values between the local ligands with PDB IDs were calculated with the affirmation of the docked ligand used for docking protocol validation. The docking protocol is considered to be remarkable and can be utilized for the further docking process.

### 4.5. Molecular Docking

The docking simulation was performed to examine the authoritative mode in the active site of different proteins: PPARα: 1K7L [44], PPARβ: 1GWX [45], and PPARγ: 3DZY [46]. The data regarding the experimental resolution of each protein could be consulted in the Protein Data Bank web site. The 3D structures of the compounds or ligands were subjected to a geometrical optimization at ACD/ChemSketch/3D. All optimized conformations were confirmed to have the lowest potential energy.

### 4.6. AutoDock 4.2.6

Molecular docking for the set of optimized ligands was performed using the Auto Dock program version 4.2.6 [47]. Auto Dock combines a quick energy assessment through pre-calculated grids of affinity conceivable outcomes with medicinal chemistry research, thus allowing grouping of calculations to determine the appropriate binding positions for a ligand on a given macromolecule. This program solidifies the van der Waals attraction potential, geometric collision, screened electrostatic potential, and Lazaridis–Karplus de-solvation energy into the score. Thus, all nuclear docking results shown in this study are the global docking scores. Within the course of action of the proteins for docking recreation, the water atoms, cofactors, and particles were disallowed from each X-ray crystallographic structure. The polar hydrogen particles of the proteins were included, the atomic charges were computed using the Gasteiger technique, and the nonpolar hydrogen particles were combined. Finally, the chemical was treated as a rigid body. Atomic docking calculations were performed inside the active site of each protein. Nuclear docking frequently requires a user-defined docking space in which the conceivable ligand definitive conformations are examined. A small search space can produce an insufficient number of conformations, although a generously expansive space may deliver various inconsequential interaction stances. Consequently, in a perfect world, limited docking look space is significant to the success of ligand-protein coupling. The default box size can be calculated using experimentally resolved protein–ligand complex structures. To start with, an initial box is constructed to enclose the ligand, and then the size of the box is increased in random directions to ensure that the minimum length in any dimension is at least 22.5 Å. The grid maps of interaction energy for various atom types with each macromolecule were calculated by the auxiliary program Auto Grid choosing a grid box centered at: (−17, −14, −4) for 1K7L, and (−11, 19.5, 15) for 3DZY, with dimensions of 40 × 40 × 40 Å around the active site, and a grid point spacing of 0.375 Å. All these conditions are sufficiently to include the most important residues of each enzyme. The docking searches for the best orientations of the molecules to the active site of each protein were per formed using the Lamarckian genetic algorithm (LGA) [48]. The LGA protocol applied a population size of 2000 individuals, while 2,500,000 energy valuations were used for the 200 LGA runs. The leading docking complex arrangements (postures) were analysed based on the potential intermolecular interactions (ligand/enzyme), such as hydrogen bonding, hydrophobic interactions, and cation–π, π–π stacking.

### 4.7. AutoDock Vina

Vina utilized the progressed angle optimization technique in the local optimization strategy. It is the improved version with more docking accuracy, including a new scoring function, and has efficient optimization [49]. The molecular docking studies were performed to predict and understand binding of macromolecules and small molecules efficiently. The AutoDock apparatus was used to determine the polar hydrogen in the protein structure. The grid box was utilized for the arrangement of the framework outline, and the size measure was set to 30 × 30 × 30 XYZ focuses. Encourage, an adaptation record that contained protein, ligand, and grid data, was arranged to allow docking investigation. AutoDock Vina provides nine authoritative modes for each ligand, and the poses are positioned in agreement with the binding affinity. Compounds with the most favorable and most noteworthy binding affinity were chosen for further investigation.

### 4.8. Dock6

Hydrogen atom orientations were optimized using the sander module in AMBER16 for a maximum of 100 cycles of minimization, with heavy restraints (1000.0 kcal mol–1 Å–2) on all non-hydrogen atoms [50]. DOCK6 required the MOL2 format of the protein, and ligand coordinates were extricated and saved.

### 4.9. Docking Visualization

The abovementioned docking outcomes were observed on UCSF Chimera version 1.14 (RRID: SCR_004097). Only one compound (Table 13) was selected for the visualization among six compounds. The two-dimensional interactions of the complex protein-ligand structure, including the hydrogen bonds, hydrophobic interactions, and bond lengths, were analysed using LigPlot+ (RRID: SCR_018249) for high-affinity bonds.

### 4.10. Toxicity Screening 

Toxicity screening was performed using the ProTox-II webserver https://tox-new.charite.de/protox_II/ (accessed on 16 April 2021), and the test compounds were arranged in SMILES coordinates. At that point, the input on the ProTox-II webserver runs the calculation program for toxic quality. The toxicity level was determined using the LD_50_ (mg/kg unit).

### 4.11. ADMET Prediction

An in silico ADME investigation showed the process of selecting compounds by determining the basic pharmacokinetic parameters such as absorption, distribution, metabolism, and excretion [51]. The PreADMET https://preadmet.bmdrc.kr/adme/ (accessed on 16 July 2021) online software was used to estimate the pharmacokinetic properties of the selected compounds, while the SwissADME tool http://www.swissadme.ch/ (accessed on 19 July 2021) was used to calculate the drug-likeness based on the Lipinski’s and Veber’s rules. The different properties of selected compounds were as follows. The Caco-2 cells differentiate to form tight junctions between cells in order to enable paracellular movement of compounds across the monolayer (Caco-2 cell model). Human intestinal absorption (HIA) is one of the most important ADME properties and involves the transport of drugs to their targets. Plasma protein binding (PPB) refers to the degree to which drugs attach to proteins within the blood. Moreover, the blood-brain barrier (BBB) prevents the brain uptake of most drugs. Meanwhile, an ADME property based on Madin-Darby Canine Kidney (MDCK) cell line involves the intestinal drug absorption of small molecules and is correlated to human intestinal permeability (MDCK cell permeability); furthermore, skin permeability was evaluated.

### 4.12. Molecular Dynamics Simulation

The Gromacs 5.1.2 program (RRID:SCR_014565) was utilized to perform molecular dynamic (MD) simulations of the PPARα-DPP or eicosapentaenoic corrosive (control) complex structures. PPARα atomic drive field parameters were written from CHARMM36 [52], which is an all-atom drive lipid force field, while the compound DPP or eicosapentaenoic corrosive (control) atomic drive field parameters were obtained from the Gromos54a7 force field using the Automated Topology Builder ATB, https://atb.uq.edu.au/index.py (accessed on 27 April 2021), which were then converted into the GROMACS file format. Initially, energy minimization was executed by employing a steep descent strategy of 50,000 steps to achieve steady compliance. After minimization, the isobar isothermal ensembles (NPT) and canonical ensembles (NVT) were applied. Individually, a consistent weight of 1 atm per 100 ps and a consistent temperature of 300 K for NPT and a consistent temperature of 300 K for NVT were maintained over a period of 100 ps. The generation MD runs were performed for 10 ns, maintaining the temperature at 300 K and the weight at 1 bar. The RMSD, root mean square change (RMSF), and the distance between PPARα and DPP or eicosapentaenoic acid were calculated after the runs. Similarly, PPARɣ and DPP were also calculated. These parameters were outlined using the Gnuplot program (RRID:SCR_008619).

### 4.13. Chemicals and Reagents

DPP and dimethyl sulfoxide (DMSO) were purchased from Sigma-Aldrich (St. Louis, MO, USA). Antibody against TFIIB (sc-271736) was purchased from Santa Cruz Biotechnology (Santa Cruz, CA, USA). PPARγ (PA3-821A) and PPARα (ab24509) antibodies were obtained from Thermo Fisher Scientific (Waltham, MA, USA) and Abcam Inc. (Cambridge, MA, USA), respectively.

### 4.14. Cell Culture and Cell Viability Assay

Rat liver (Ac2F) cells were obtained from the American Type Culture Collection (Rockville, MD, USA) and cultured in Dulbecco’s Modified Eagle Medium (Gyeongsan-si, Daegu, South Korea) containing 2 mM l-glutamine, 100 µg/mL streptomycin (HyClone, Logan, UT, USA), and 10% heat-inactivated fetal bovine serum (Gibco, Grand Island, NE, USA) at 37 °C in a humidified atmosphere containing 5% CO_2_. Cell viability was assessed using the Ez-Cytox cell viability assay kit (Daeil Lab Service Co. Ltd, Seoul, Korea). The compounds were then broken down in DMSO.

### 4.15. Transfection and Luciferase Assay

For luciferase assays, 100 µL of Ac2F cells were seeded at a density of 5 × 10^3^ cells in a 96-well plate. The cells were transfected with Lipofectamine transfection reagent (Thermo Fisher Scientific, Rockford, IL, USA) and then transfected with the PPRE-X3-TK-LUC plasmid (Dr. Christopher K. Glass, College of California, San Diego, CA, USA) and full-length human PPARα and PPARγ expression vectors (Dr. Han Geuk Seo, Konkuk College, Seoul, South Korea). After transfection for 24 h, the cells were treated with WY14643 (a PPARα agonist), rosiglitazone (a PPARγ agonist), or DPP for 5 h. Luciferase activity was measured utilizing the ONE-Glo Luciferase Assay System (Promega, Madison, WI, USA) and a luminescence plate reader (Berthold Advances GmbH & Co., Bad Wildbad, Germany).

### 4.16. Western Blot Analysis

The Ac2F cells were incubated with DPP using the abovementioned concentrations for 24 h. Cell lysates were added; 30 µg of proteins was resolved by sodium dodecyl sulfate-polyacrylamide gel electrophoresis (SDS-PAGE) using an 8–10% gel and transferred to a polyvinylidene fluoride (PVDF) membrane (Millipore, Burlington, MA, USA). The membrane was blocked with 5% skim milk for 1 h at room temperature and incubated overnight with primary antibody (1:1000 dilution), followed by incubation with horseradish peroxidase-conjugated secondary antibodies (Santa Cruz Biotechnology). Counter-acting agent labeling was identified using WesternBright peroxide solution (Advansta, CA, USA) and Davinci-Chemi CAS-400 (Davinch-K, Seoul, Korea), in accordance with the manufacturers’ instructions.

### 4.17. Statistical Analysis

Statistical analysis was performed using GraphPad Prism 5 (GraphPad Software, La Jolla, CA, USA). Student’s t-test was used to determine the differences between the two groups. Statistical significance was set at *p* < 0.05.

## 5. Conclusions

In conclusion, we demonstrated that DPP has a high binding affinity and can activate PPARα and PPARγ without causing toxicity. Concomitant with the in silico results, we confirmed that DPP significantly increased the transcriptional activities of both PPARα and PPARγ and activated PPARα and PPARγ in Ac2F liver cells. To the best of our knowledge, this is the first in vitro and in silico study to report that DPP can act as a dual agonist of PPARα/γ. The findings of this study suggest that the CGJ-specific compound DPP could be a novel PPARα/γ dual agonist; however, further studies are warranted to examine its therapeutic potential in various metabolic disorders linked with PPARα/γ dysregulation.

## Figures and Tables

**Table 1:** Volatile compounds only present in CGJ fermented with pure *B. subtilis*.

Serial No	Volatile Compounds	NF	F
	**Ketones**		
1–181	1,3-Diphenyl-2-propanone	−	+
2	2,3-Butandione	−	+
3	2,6-Dihydroxyacetophenone	−	+
4	3-Penten-2-one	−	+
5	Pyrovalerone	−	+
	**Alcohols**		
6	1-Dodecanol	−	+
7	1-Octanol	−	+
8	2,3-Butanediol	−	+
9	2,5-Dimethyl-3-hexanol	−	+
10	3-Methyl-2-butanol	−	+
11	Benzyl alcohol	−	+
12	Ethanol	−	+
	**Acids and esters**		
13	2-Methyl-decanoic acid	−	+
14	2-Methyl-hexanoic acid	−	+
15	3-Methyl-butanoic acid	−	+
16	3-Methyl-pentanoic acid	−	+
17	Butanoic acid, 3-hydroxy-ethyl ester	−	+
18	Formic acid, 1-methylpropyl ester	−	+
19	9,12-Octadecadienoic acid methyl ester	−	+
20	Benzeneacetic acid, ethyl ester	−	+
21	Octanoic acid ethyl ester	−	+
	**Miscellaneous**		
22	2,4-Di-*tert*-butylphenol	−	+
23	2-Methoxy-4-(2-propenyl)phenol	−	+
24	3,4-Dihydroxyphenylglycol	−	+
25	*trans*-Calamenene	−	+
	**Pyrazine**		
26	2,5-Dimethyl-pyrazine	−	+
27	2-Butyl-3,5-dimethyl-pyrazine	−	+
28	3-Ethyl-2,5-dimethyl-pyrazine	−	+
29	Tetramethyl-pyrazine	−	+
	**Aldehyde**		
30	Acetaldehyde	−	+
31	Alpha-ethylidene-benzeneacetaldehyde	−	+
32	Nonanal	−	+
33	Piperonal	−	+
34	Benzeneacetaldehyde	−	+
	**Aromatic compounds**		
35	4-(Nonafluoro-*tert*-butyl) nitrobenzene	−	+

−: Absence, +: presence, NF: non fermented, F: fermented.

**Table 2:** In silico docking simulation of active volatile components of fermented soybean CGJ with PPARs.

Component	PPARα			PPARβ/δ			PPARγ		
	Vina	AD4	Dock6	Vina	AD4	Dock6	Vina	AD4	Dock6
Control	−6.7	−7.81	−35.45	−7.8	−7.44	−41.006	−6.6	−7.89	−32.666
1,3-Diphenyl-2-propanone	−8.8	−8.14	−30.4204	−7.5	−7.36	−34.8391	−7.0	−7.53	−36.0086
2,4-Di-*tert*-butylphenol	−6.8	−7.17	−28.6838	−7	−7.03	−27.4858	−5.6	−6.74	−28.516
4-(Nonafluoro-*tert*-butyl) nitrobenzene	−6.1	−5.25	−25.5059	−7.7	−5	−24.3869	−6.1	−4.86	−28.3937
9,12-Octadecadienoic acid methyl ester	−6.3	−8.18	−26.8669	−6.9	−7.34	−28.9908	−6.3	−7.83	−27.3687
Pyrovalerone	−7.5	−8.41	−124.603	−7.6	−7.75	−129.638	−6.7	−7.78	−130.539
*trans*-Calamenene	−6.8	−8.02	−29.2324	−7.6	−7.8	−28.4842	−6.5	−7.71	−27.7687

vina; Autodock vina, AD4; Autodock 4, PPARα; Peroxisome proliferator-activated receptor alpha, PPARβ/δ; Peroxisome proliferator-activated receptor beta, PPARγ; Peroxisome proliferator-activated receptor gamma.

**Table 3:** In silico docking simulation of active volatile components of fermented soybean CGJ with AMPK, LKB1 and PAR2.

Component	AMPK			LKB1			PAR2	
	Vina	AD4	Dock6	Vina	AD4	Vina	AD4	Dock6
Control	−5.7	−10.81	−132.631	−7.4	−7.22	−4.8	−8.19	−105.242
1,3-Diphenyl-2-propanone	−7.2	−10.73	−37.2376	−7.7	−8.81	−5	−7.07	−29.2367
2,4-Di-*tert*-butylphenol	−7	−6.41	−25.7078	−7.1	−6.06	−4.6	−5.49	−21.2142
4-(Nonafluoro-*tert*-butyl) nitrobenzene	−7.7	−9.65	−29.9102	−7.3	−8.48	−5.2	−6.93	−26.5166
9,12-Octadecadienoic acid methyl ester	−7.2	−3.51	−28.1999	−7.3	−4.15	−4.4	−2.89	−22.5024
Pyrovalerone	−6.4	−6.57	−25.6285	−5	−5.46	−3.4	−3.85	−21.3088
*trans*-Calamenene	−6.8	−7.19	−133.185	−6	−7.09	−4.4	−6.06	−99.2638

vina; Autodock vina, AD4; Autodock 4, AMPK; adenosine monophosphate-activated protein kinase, LKB1; liver kinase B1, PAR2; Protease activated receptor 2.

**Table 4:** In silico docking simulation of active volatile components of fermented soybean CGJ with SIRTs.

Component	SIRT1			SIRT2			SIRT3			SIRT6	
	Vina	AD4	Dock6	Vina	AD4	Dock6	Vina	AD4	Dock6	Vina	AD4
Control	−7.9	−9.23	−38.382	−11.4	−12.01	−53.2537	−5.6	−11.44	−231.781	−9.9	−8.5
1,3-Diphenyl-2-propanone	−6.9	−9.67	−34.439	−9.1	−11.58	−37.1523	−9.4	−10.59	−42.1555	5.1	0.63
2,4-Di-*tert*-butylphenol	−8.7	−7.63	−29.9781	−8.1	−7.22	−29.2715	−6.9	−6.37	−24.8486	−7.1	−7.27
4-(Nonafluoro-*tert*-butyl) nitrobenzene	−8.4	−11.65	−34.5245	−8.9	−10.87	−33.2557	−8.5	−9.31	−37.0224	−5.6	−10.36
9,12-Octadecadienoic acid methyl ester	−9	−5.63	−26.8674	−8.5	−4.71	−23.4719	−7.5	−4.14	−23.8888	−5.4	−5.15
Pyrovalerone	−7.5	−8.4	−33.1864	−7.7	−8.18	−29.2381	−6.7	−6.74	−26.5441	−6.8	−8.32
*trans*-Calamenene	−8.5	−8.78	−126.213	−8.5	−8.34	−123.2	−7.3	−7.92	−126.672	−7.3	−9.27

vina; Autodock vina, AD4; Autodock 4, SIRT; Sirtuin.

**Table 5:** The binding energy and interacting residues obtained by calculating the PPARα–DPP molecular docking scores.

Compound	Binding Energy (kcal/mol)	Inhibition Constant *K*i (μM)	Intermolecular Energy (kcal/mol)	Bonded Residues
1,3-Diphenyl-2-propanone	−8.14	1.09	−9.33	CYS276, GLN 277, SER 280, TYR 314, MET 355, LEU 456, and TYR 464

**Table 6:** The binding energy and interacting residues obtained by calculating the PPARβ-DPP molecular docking scores.

Compound	Binding Energy (kcal/mol)	Inhibition Constant *K*i (μM)	Intermolecular Energy (kcal/mol)	Bonded Residues
1,3-Diphenyl-2-propanone	−7.36	4.05	−8.37	PHE 282, CYS 285, THR 289, ILE 364, and HIS 449

**Table 7:** The binding energy and interacting residues obtained by calculating the PPARγ-DPP molecular docking scores.

Compound	Binding Energy (kcal/mol)	Inhibition Constant *K*i (μM)	Intermolecular Energy (kcal/mol)	Bonded Residues
1,3-Diphenyl-2-propanone	−7.35	3.05	−8.18	CYS 285, ARG 288, SER 289, and LEU 330

**Table 8:** Toxicity prediction based on the docking scores of active volatile components of CGJ using the ProTox-II tool.

Serial No	Volatile Compounds	Toxicity LD_50_ (mg/Kg)
1	1,3-Diphenyl-2-propanone	2000
2	2,4-Di-*tert*-butylphenol	700
3	4-(Nonafluoro-*tert*-butyl) nitrobenzene	1000
4	9,12-Octadecadienoic acid methyl ester	20,000
5	Pyrovalerone	350
6	*trans*-Calamenene	6700

**Table 9:** Druglikeness properties (Lipinski rule &Veber rule) of cheongukjang specific volatile compounds and controls.

Rules	Eicosapentaenoic Acid (PPARα)	Rosiglitazone (PPARγ)	1,3-Diphenyl-2-propanone	2,4-Di-*tert*-butylphenol	4-(Nonafluoro-*tert*-butyl) Nitrobenzene	9,12-Octadecadienoic Acid Methyl Ester	Pyrovalerone	*trans*-Calamenene	^b^ Required Parameters
^a^ MW	302.5	357.4	210.27	206.32	341.13	294.5	245.36	202.33	≤500
^a^ HBD	1	1	0	1	0	0	0	0	≤5
^a^ HBA	2	6	1	1	11	2	2	0	≤10
^a^ LogP	5.6	3.1	3.1	4.9	5.6	6.9	3.8	5.1	2–5
^a^ Rot bonds	13	7	4	2	1	15	5	1	≤10

^a^ Obtained from PubChem database: MW: molecular weight, HBD: number of hydrogen donors, HBA: number of hydrogen acceptors, Rot bonds: number of rotatable bonds, ^b^ Required parameters necessary to achieve suitable physiochemical properties mostly important for BBB permeability.

**Table 10:** In silico ADME profiling of CGJ, a volatile compound acquired from the PreADMET server.

Compounds	Absorption				Distribution	
	Human Intestinal Absorption (HIA %)	Caco-2 Cell Permeability (nm s^−1^)	MDCK Cell Permeability (nm s^−1^)	Skin Permeability (logKp, cm h^−1^)	Plasma Protein Binding (%)	Blood–Brain Barrier Penetration (Cbrain/Cblood)
Eicosapentaenoic acid (PPARα)	97.940242	30.0843	76.2528	−0.566312	100	6.59907
Rosiglitazone (PPARγ)	97.451401	28.616	2.07646	−3.44324	91.095751	0.012398
1,3-Diphenyl-2-propanone	100	54.5905	209.282	−1.6951	90.912956	1.75109
2,4-Di-*tert*-butylphenol	100	44.8684	116.431	−0.739126	100	10.0918
4-(Nonafluoro-*tert*-butyl) nitrobenzene	96.809262	22.6752	3.91751	−0.758863	100	1.01857
9,12-Octadecadienoic acid methyl ester	100	47.1151	67.0846	−0.538746	100	13.8625
Pyrovalerone	100	57.4649	157.355	−1.55534	76.382571	1.46433
*trans*-Calamenene	100	23.4586	60.7588	−0.761116	100	11.9795

**Table 11:** In silico metabolism profile of CGJ, a volatile compound obtained from the PreADMET server.

Compounds	CYP2C19 Inhibition	CYP2C9 Inhibition	CYP2D6 Inhibition	CYP2D6 Substrate	CYP3A4 Inhibition	CYP3A4 Substrate
Eicosapentaenoic acid (PPARα)	Inhibitor	Inhibitor	Non	Non	Inhibitor	Non
Rosiglitazone (PPARγ)	Non	Inhibitor	Non	Non	Non	Weakly
1,3-Diphenyl-2-propanone	Inhibitor	Inhibitor	Non	Non	Inhibitor	Weakly
2,4-Di-*tert*-butylphenol	Non	Inhibitor	Non	Non	Inhibitor	Substrate
4-(Nonafluoro-*tert*-butyl) nitrobenzene	Non	Inhibitor	Non	Non	Inhibitor	Substrate
9,12-Octadecadienoic acid methyl ester	Inhibitor	Inhibitor	Non	Non	Inhibitor	Non
Pyrovalerone	Non	Non	Inhibitor	Weakly	Non	Substrate
*trans*-Calamenene	Inhibitor	Inhibitor	Non	Non	Inhibitor	Substrate

**Table 12:** Hydrogen bond donor and acceptor residues obtained from the molecular dynamic simulation (PPARα).

Donor	Hydrogen	Acceptor
GLN277 NE2	GLN277 HE21	469 O1
HIS440 NE2	HIS440 HE2	469 O1

**Table 13:** Name, molecular weight, and uses of the selected CGJ specific volatile compounds.

Serial No	Compound Name	IUPAC Name	Molecular Weight (Da)	Uses
1	1,3-Diphenylacetone	1,3-diphenylpropan-2-one	210.27	Food additive, flavor
2	2,4-Di-*tert*-butylphenol	2,4-ditert-butylphenol	206.32	Making chemicals
3	4-(Nonafluoro-*tert*-butyl) nitrobenzene	1-[1,1,1,3,3,3-hexafluoro-2-(trifluoromethyl)propan-2-yl]-4-nitrobenzene	341.13	Industrial application
4	9,12-Octadecadienoic acid methyl ester	methyl octadeca-9,12-dienoate	294.5	Personal care, cosmetics
5	Pyrovalerone	1-(4-methylphenyl)-2-pyrrolidin-1-ylpentan-1-one	245.36	manufacturing of drugs
6	*trans*-Calamenene	(1S,4R)-1,6-dimethyl-4-propan-2-yl-1,2,3,4-tetrahydronaphthalene	202.33	Preservative, Insecticidal

## Supplementary Materials

The supplementary materials are available online at https://www.mdpi.com/article/10.3390/ijms221910884/s1.
