# Supplementary material for: Cheonggukjang-Specific Component 1,3-Diphenyl-2-Propanone as a Novel PPARα/γ Dual Agonist: An In Vitro and In Silico Study
Source: Int J Mol Sci. 2021 Oct 8;22(19):10884. doi: 10.3390/ijms221910884 (PMC8509681; doi:10.3390/ijms221910884)
Supplement: Supplementary file 1 [file ijms-22-10884-s001.zip › ijms-1393558-Supplementary.pdf]

**Supplementary Materials:**

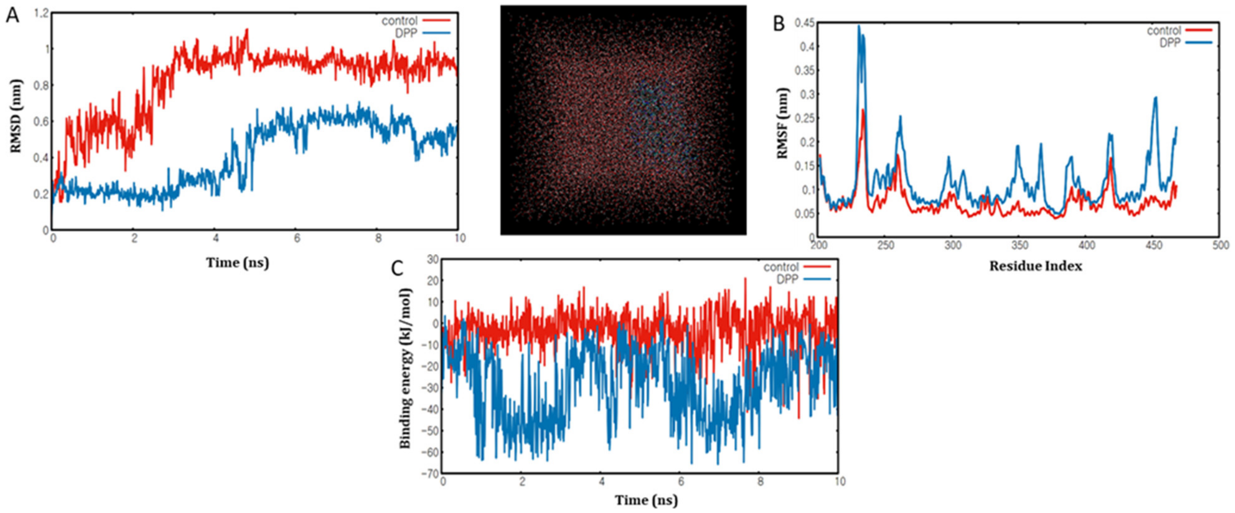


**Figure S1.** MD simulation analyses (**A**) Root mean square deviation (RMSD) plot of PPARγ Rosiglitazone (Red) and 1,3 diphenyl 2 propanone (Blue) complex at10 ns of molecular dynamic simulation. (**B**) The RMSF plot of the backbone heavy atoms of the Rosiglitazone (Red) and 1,3 diphenyl 2 propanone (Blue) complex. (**C**) Binding energy plot of Rosiglitazone (Red) and 1,3 diphenyl 2 propanone (Blue) complex at10 ns.

**Table S1**. Hydrogen bond donor and acceptor residues from the molecular dynamic simulation (PPARγ)

| Donor | Hydrogen | Acceptor |
|---|---|---|
| ARG288NE | ARG288HE | EQPX478O1 |
| ARG288NH2 | ARG288HH21 | EQPX478O1 |
| SER289OG | SER289HG1 | EQPX478O1 |
